# Supplementary material for: Novel Strigolactone Mimics That Modulate Photosynthesis and Biomass Accumulation in Chlorella sorokiniana
Source: Molecules. 2023 Oct 12;28(20):7059. doi: 10.3390/molecules28207059 (PMC10609326; doi:10.3390/molecules28207059)
Supplement: Supplementary file 1 [file molecules-28-07059-s001.zip › molecules-2630909-supplementary.pdf]

Supplementary Material

Novel strigolactone mimics that modulate photosynthesis and biomass accumulation in *Chlorella sorokiana*

by Daria Gabriela Popa, Florentina Georgescu, Florea Dumitrascu, Sergiu Shova, Diana Constantinescu-Aruxandei, Constantin Draghici, Lucian Vladulescu, Florin Oancea

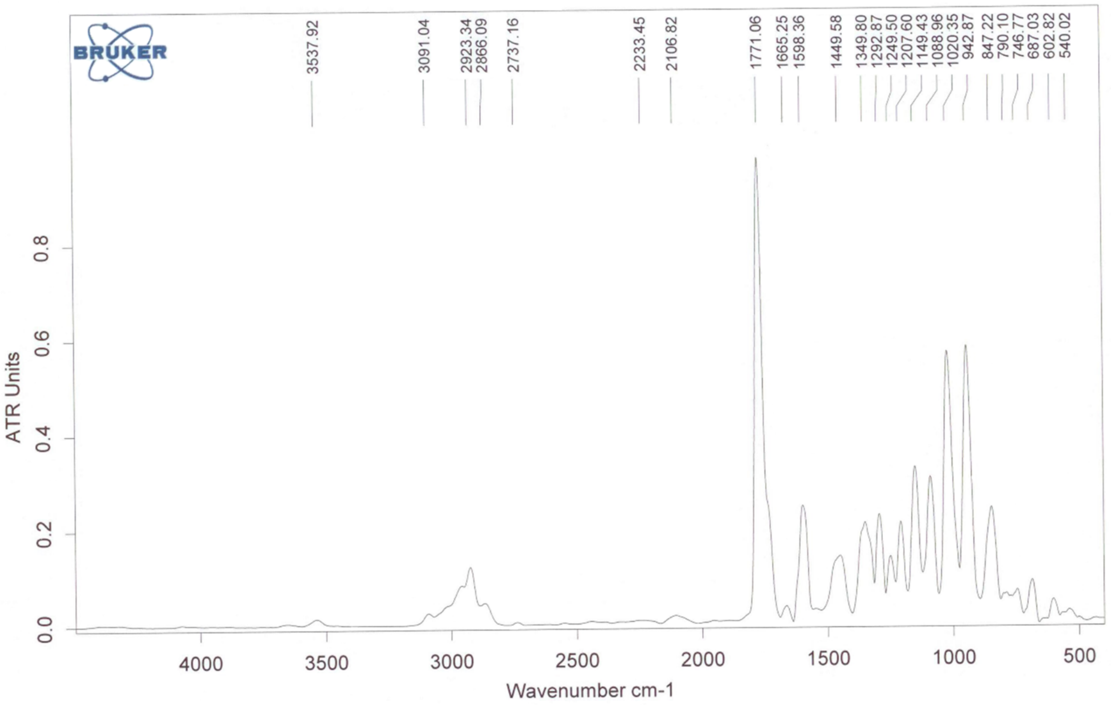

(a)

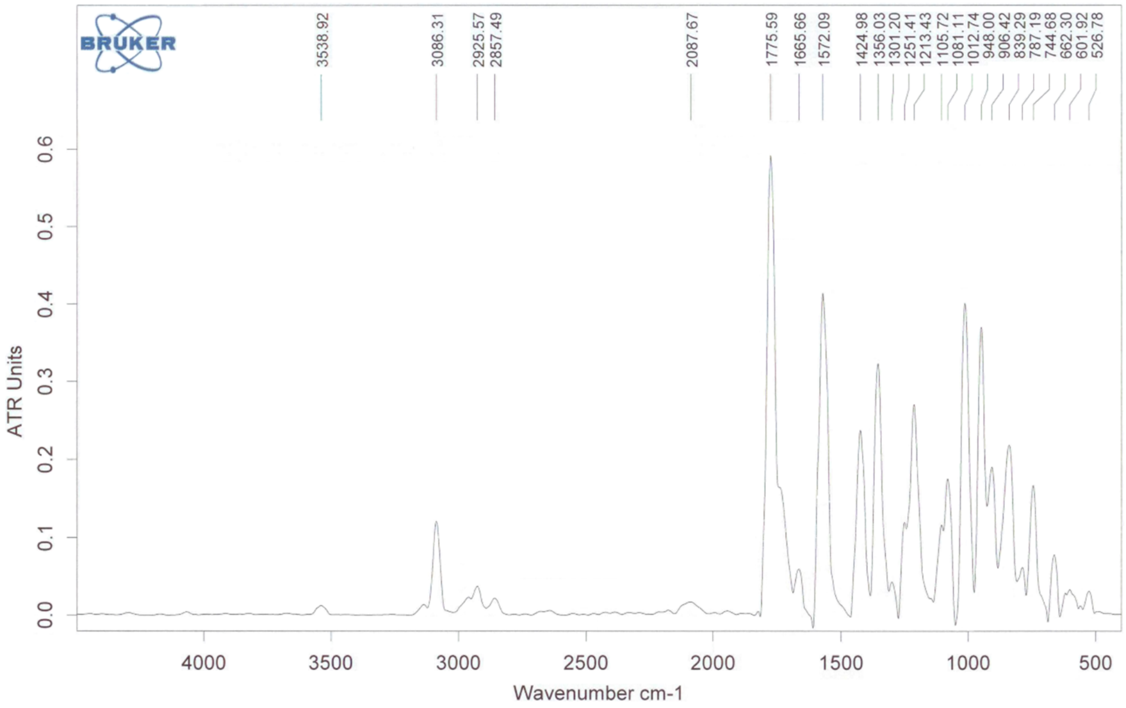

(b)

Figure S1. IR spectrum of (a) compound **3** (SL-F1) and (b) compound **4** (SL-F2)

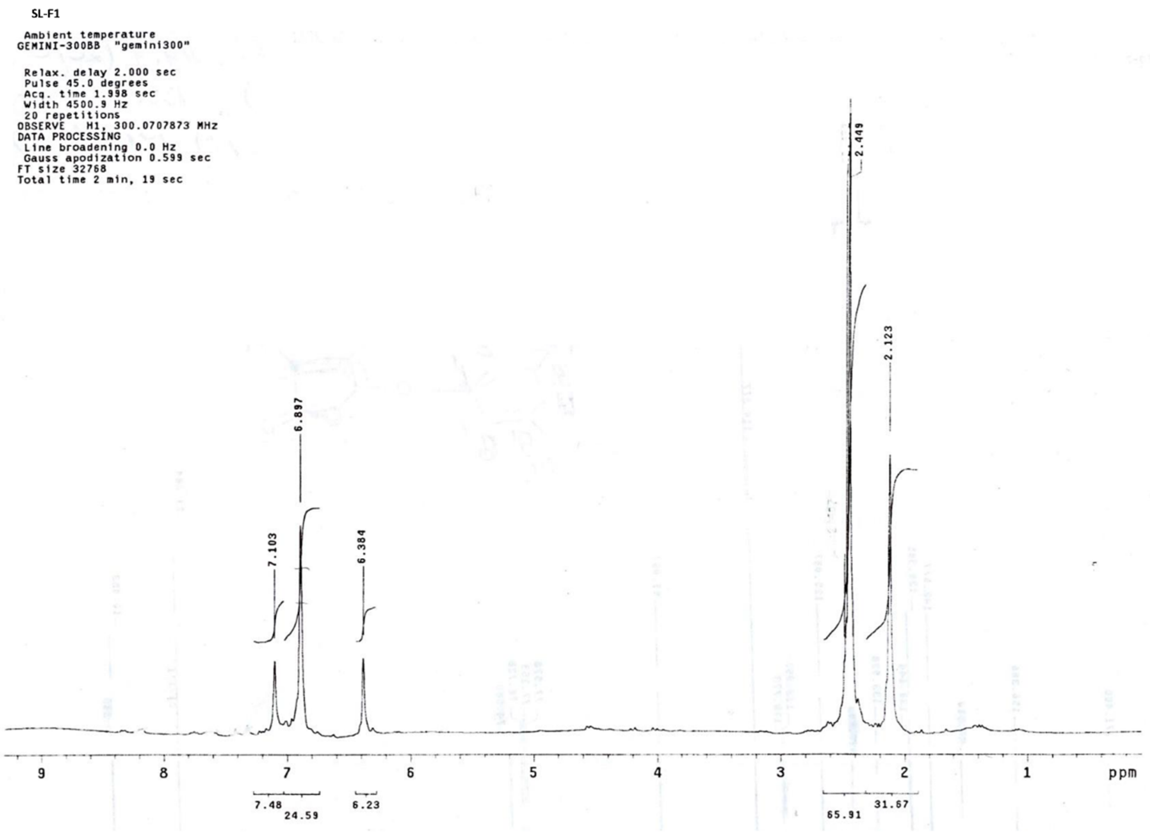

(a)

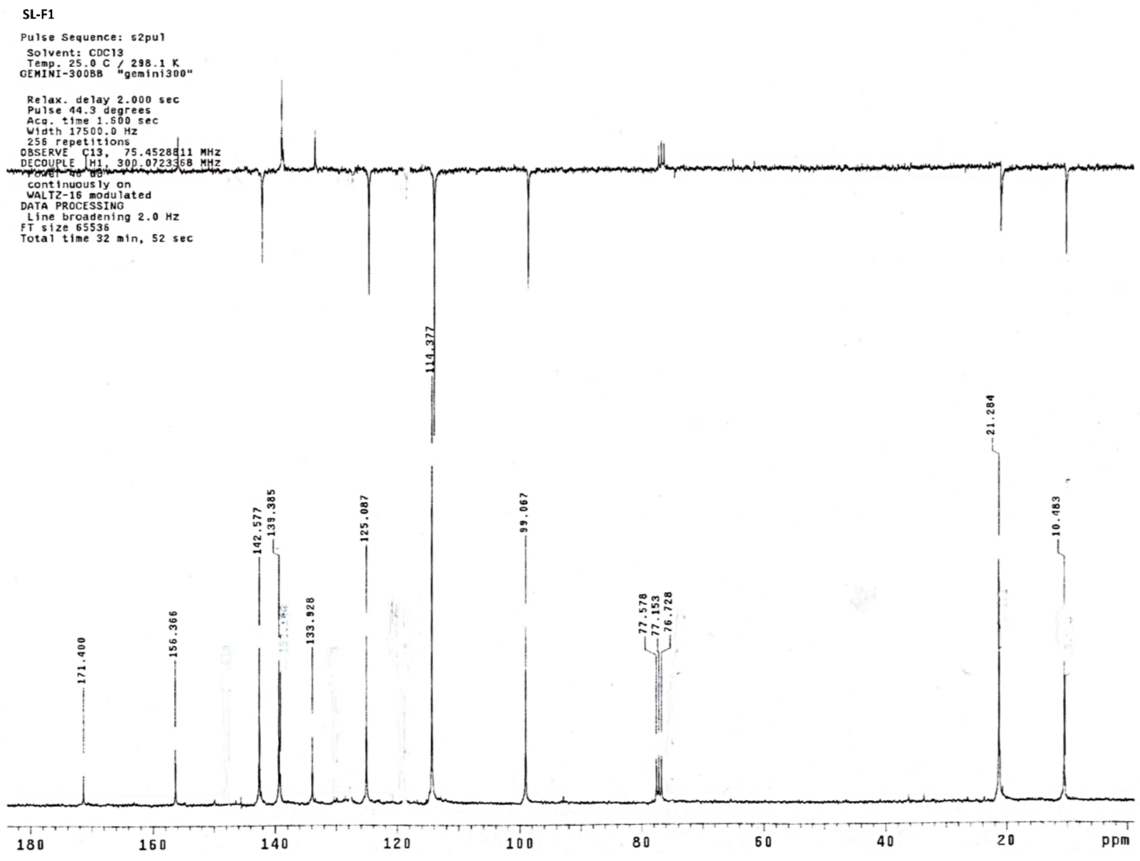

(b)

Figure S2. (a) The ^{1}H NMR spectrum for compound **3** (SL-F1), recorded in $CDCl_3$; (b) The ^{13}C NMR spectrum for compound **3** (SL-F1), recorded in $CDCl_3$.

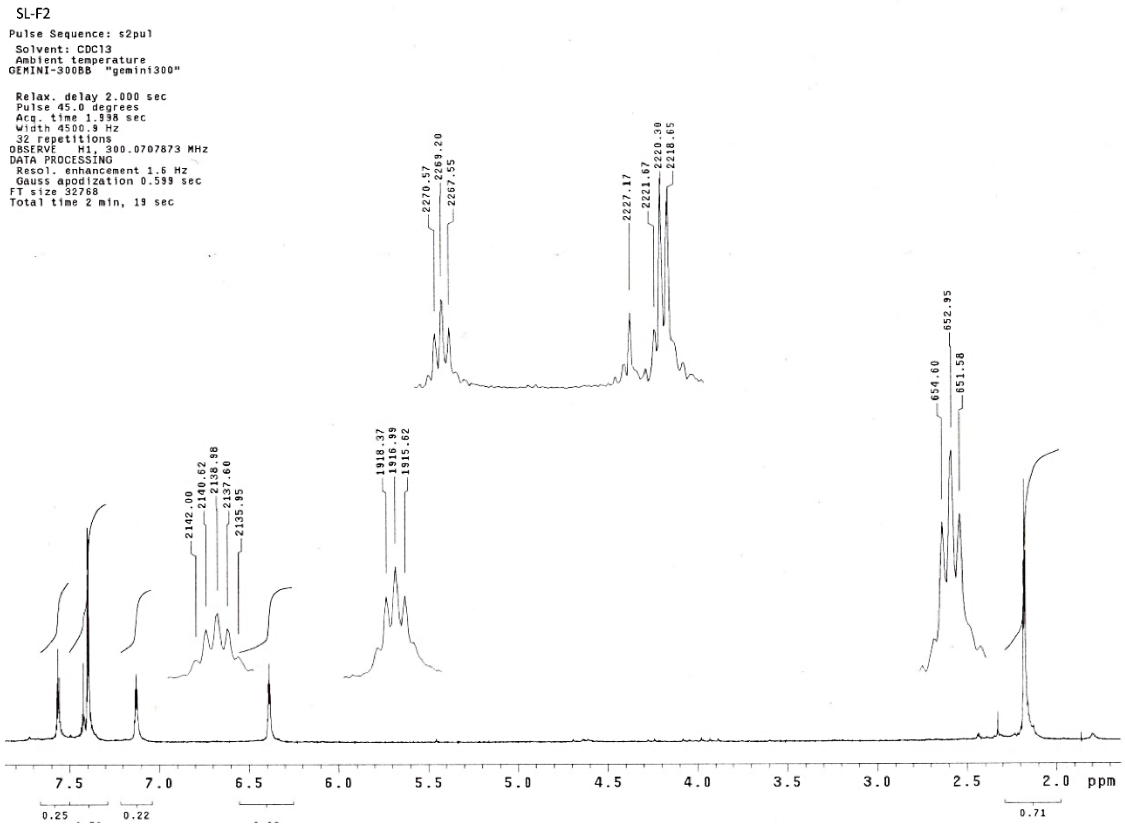

(a)

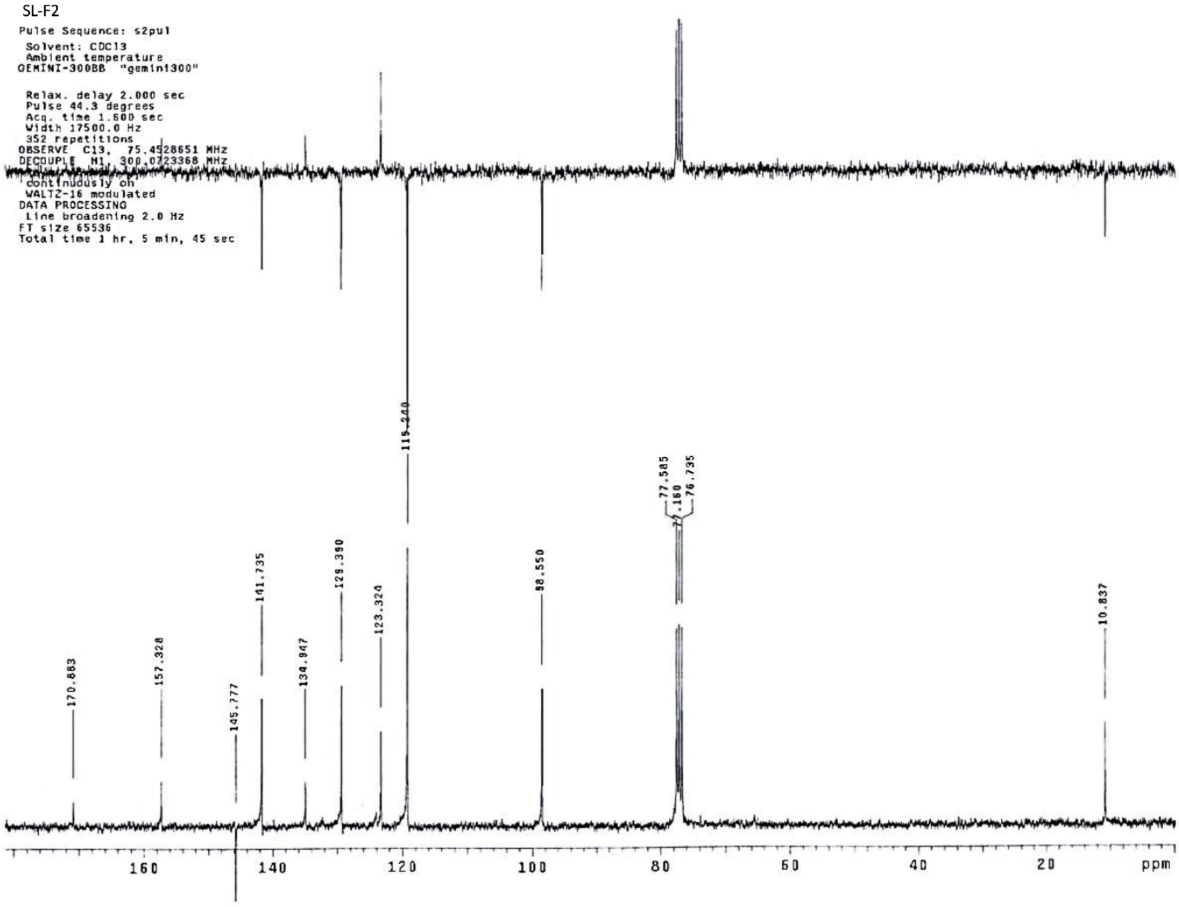

(b)

Figure S3. (a) The ^{1}H NMR spectrum for compound **4** (SL-F2) recorded in $CDCl_3$; (b) The ^{13}C NMR spectrum for compound **4** (SL-F2) recorded in $CDCl_3$.

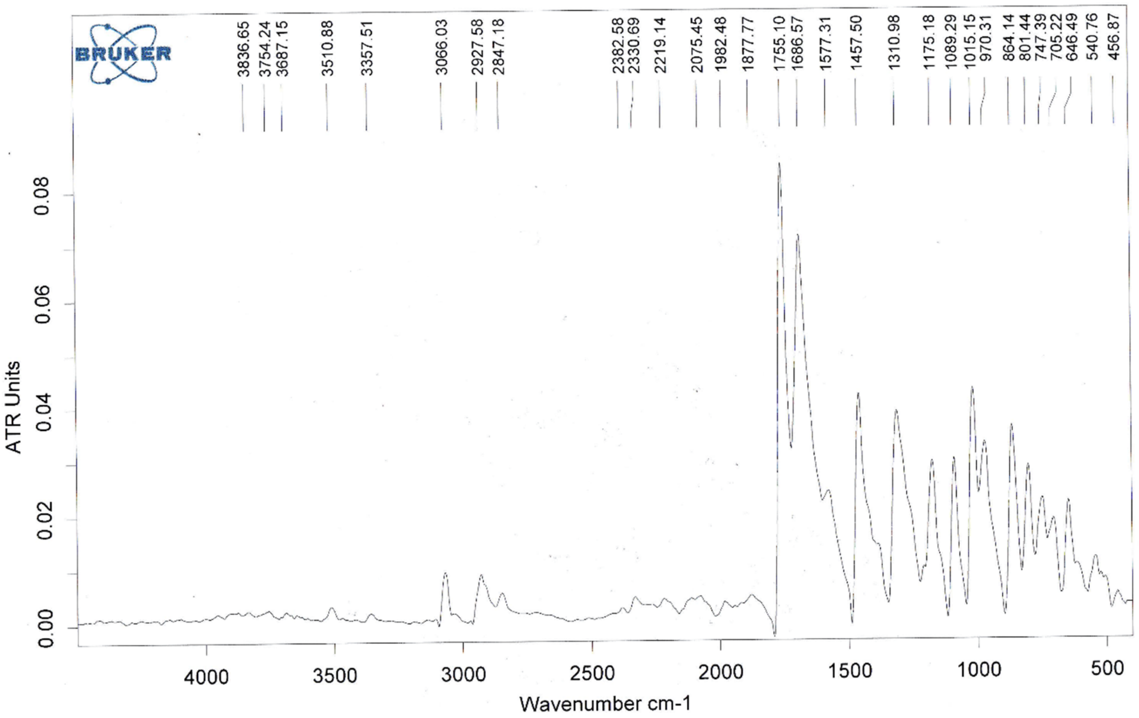
BRUKER
3836.65
3754.24
3687.15
3510.88
3357.51
3066.03
2927.58
2847.18
2382.58
2330.69
2219.14
2075.45
1982.48
1877.77
1755.10
1686.57
1577.31
1457.50
1310.98
1175.18
1089.29
1015.15
970.31
864.14
801.44
747.39
705.22
646.49
540.76
456.87
ATR Units
0.00
0.02
0.04
0.06
0.08
4000
3500
3000
2500
2000
1500
1000
500
Wavenumber cm-1

(a)

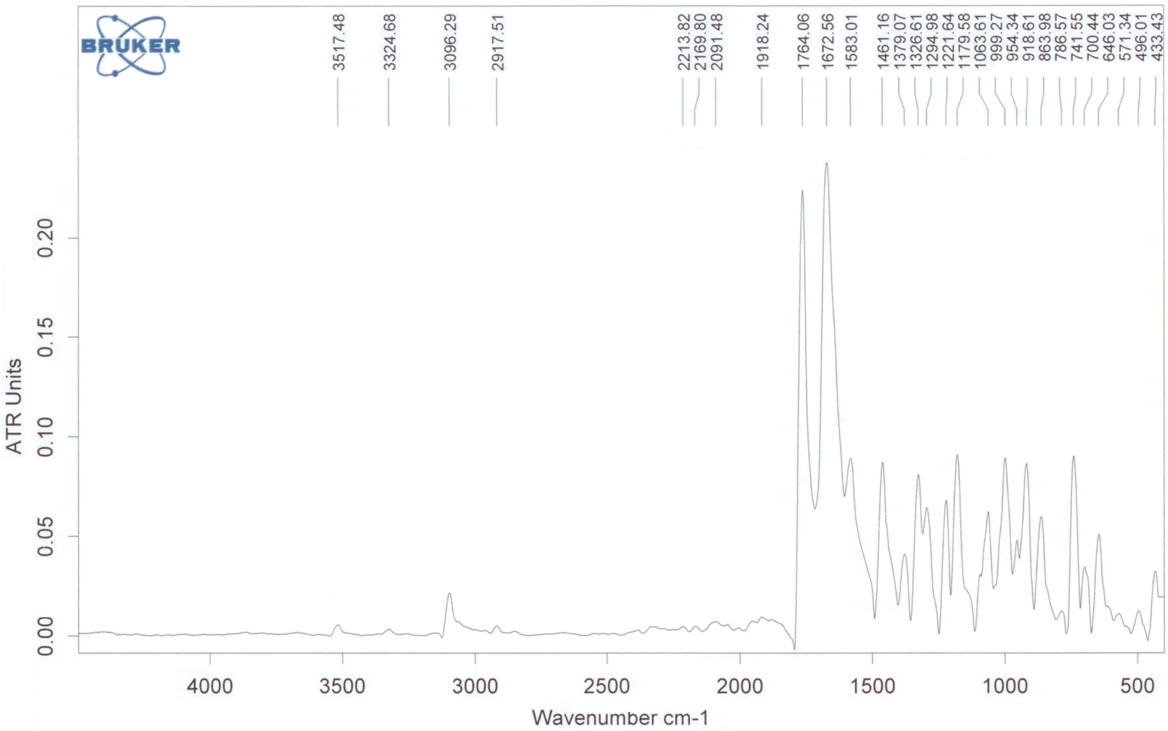
BRUKER
3517.48
3324.68
3096.29
2917.51
2213.82
2169.80
2091.48
1918.24
1764.06
1672.56
1583.01
1461.16
1379.07
1326.61
1294.98
1221.64
1179.58
1063.61
999.27
954.34
918.61
863.98
786.57
741.55
700.44
646.03
571.34
496.01
433.43
ATR Units
0.00
0.05
0.10
0.15
0.20
4000
3500
3000
2500
2000
1500
1000
500
Wavenumber cm-1

(b)

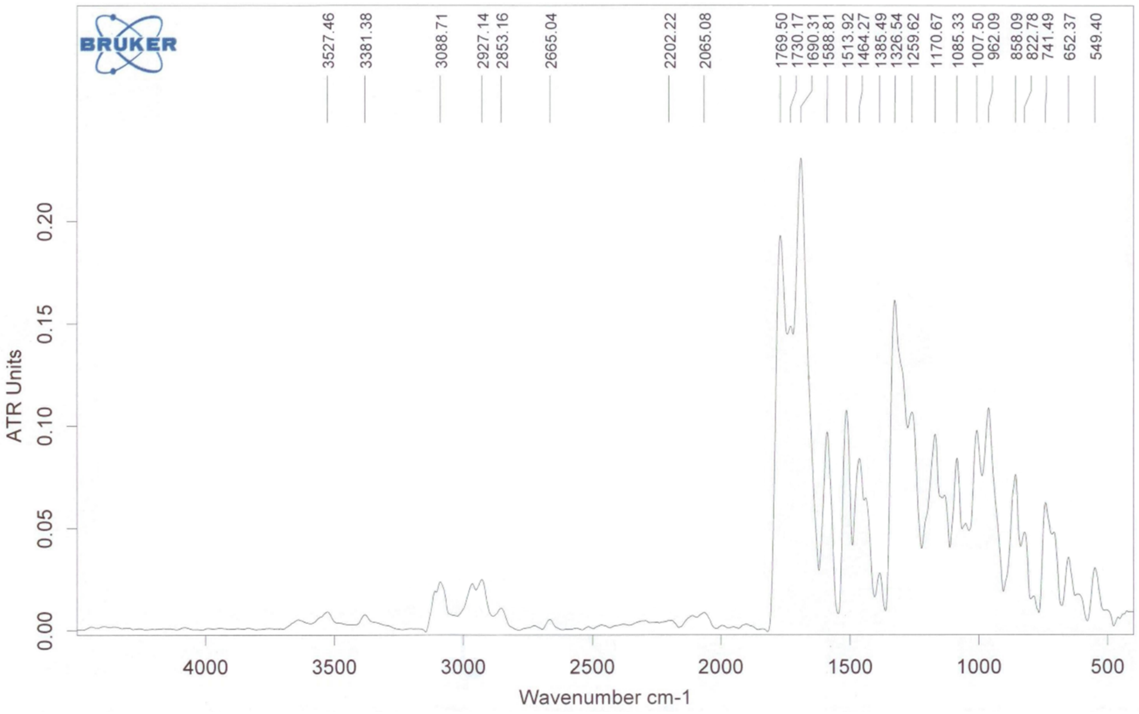

(c)

Figure S4. (a) IR spectrum of (a) compound **7** (SL-F3); (b) IR spectrum of (a) compound **9** (SL-F5); (c) IR spectrum of (a) compound **11** (SL-F6).

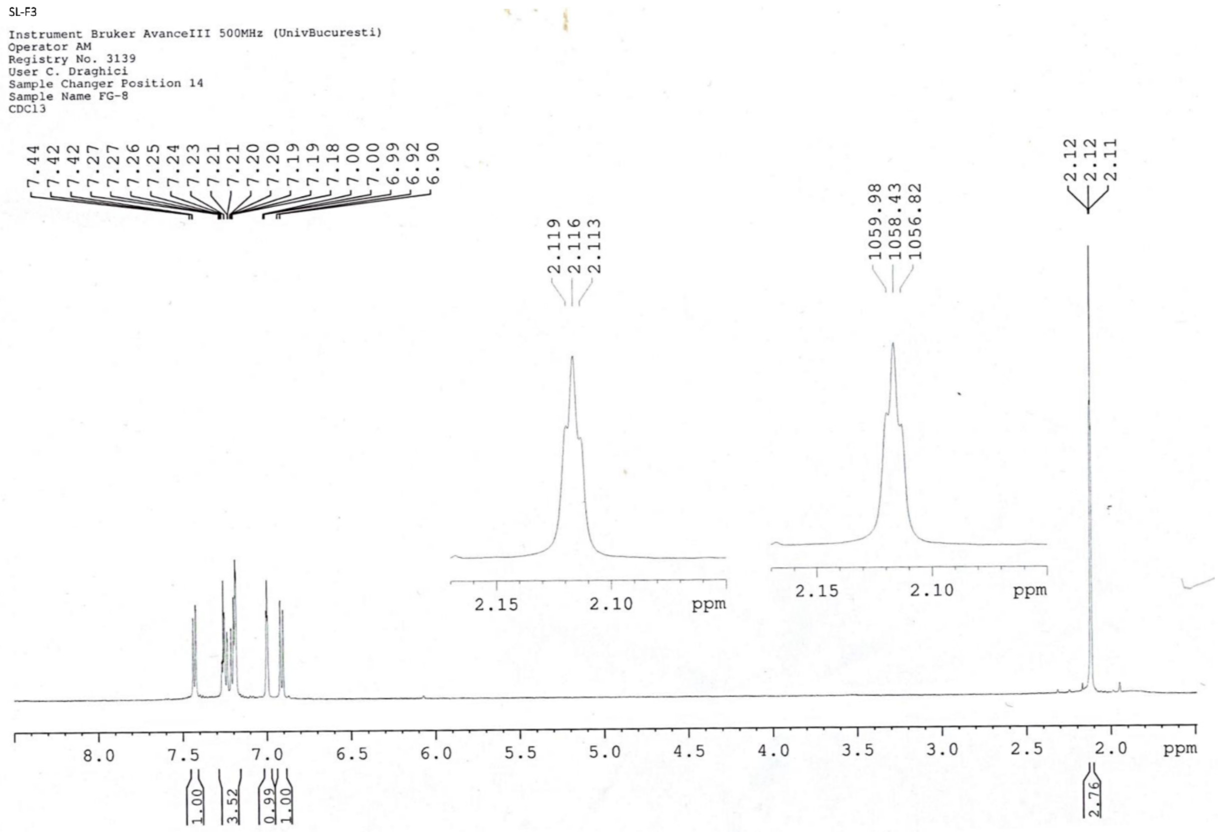

(a)

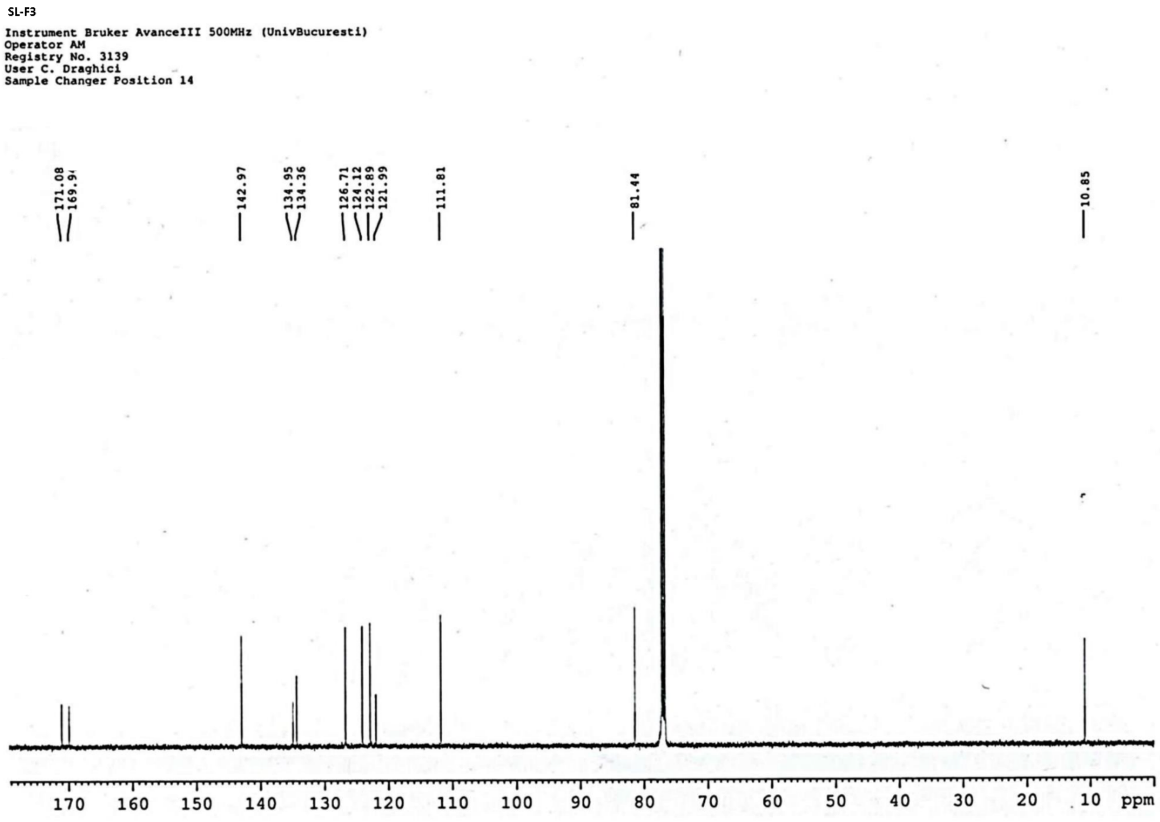

(b)

Figure S5. (a) The ^{1}H NMR spectrum for compound **7** (SL-F3) recorded in $CDCl_3$; (b) The ^{13}C NMR spectrum for compound **7** (SL-F3) recorded in $CDCl_3$.

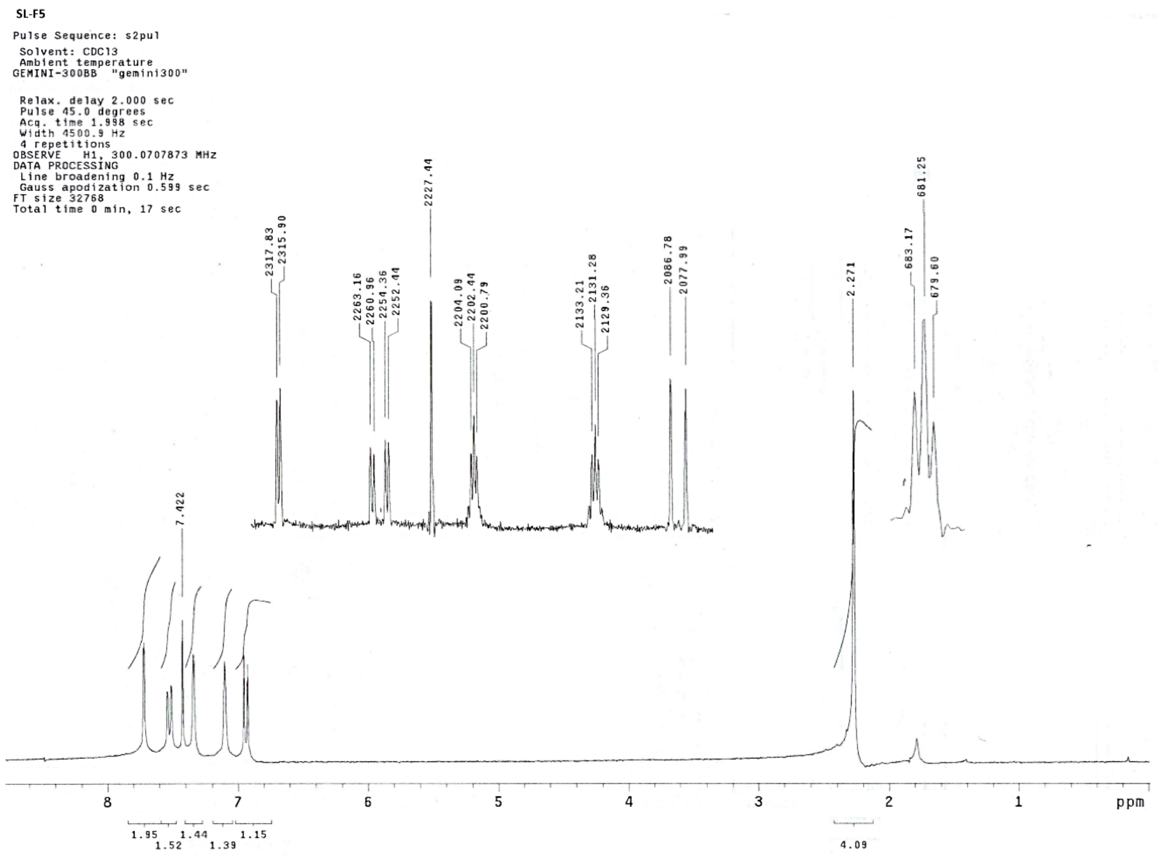

(a)

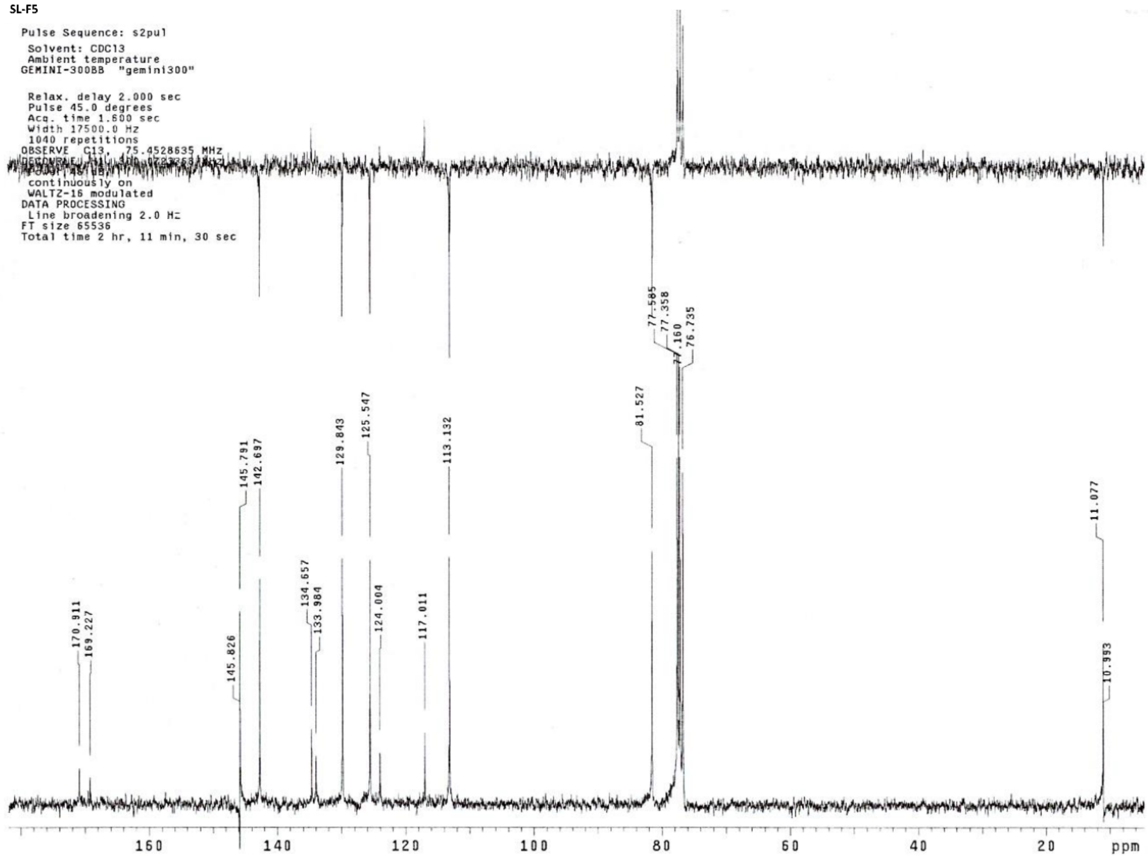

(b)

Figure S6. (a) The ^{1}H NMR spectrum for compound **9** (SL-F5) recorded in $CDCl_3$. (b) The ^{13}C NMR spectrum for compound **9** (SL-F5) recorded in $CDCl_3$.

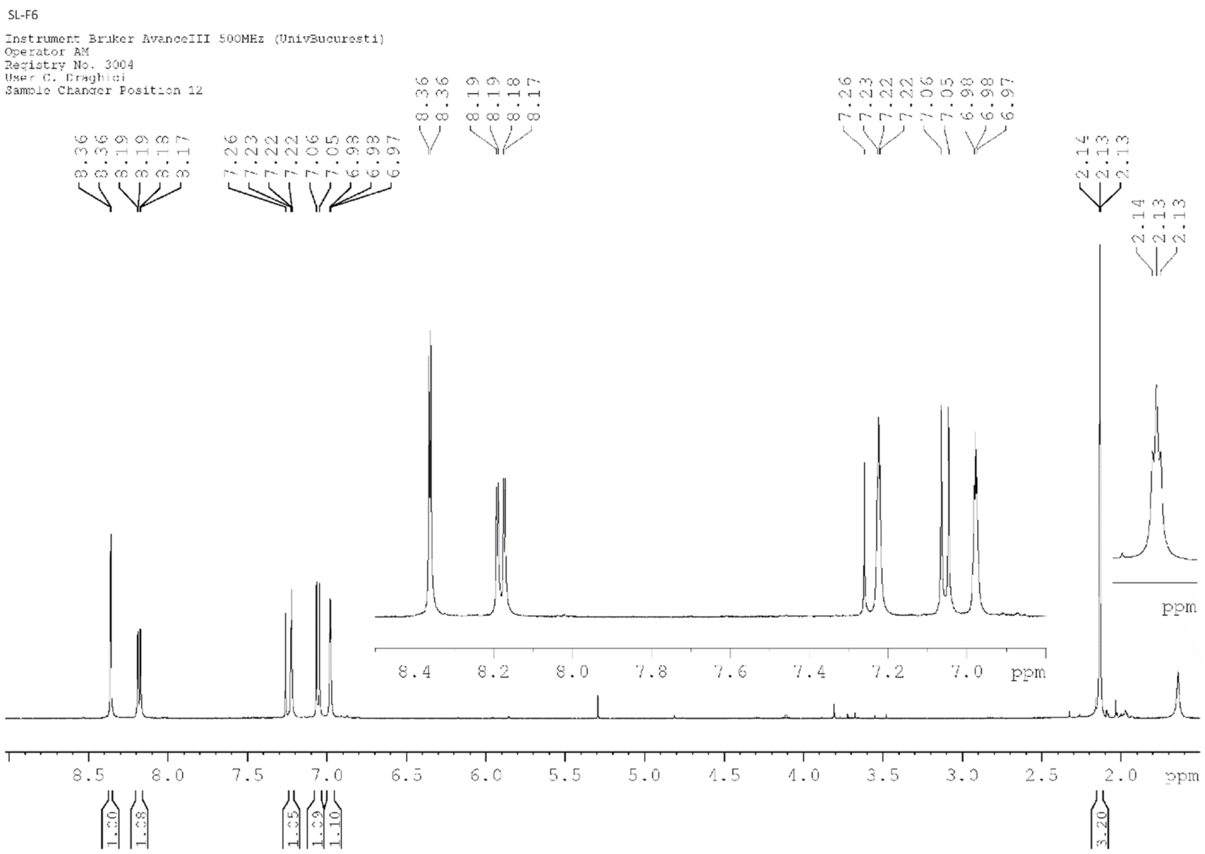

(a)

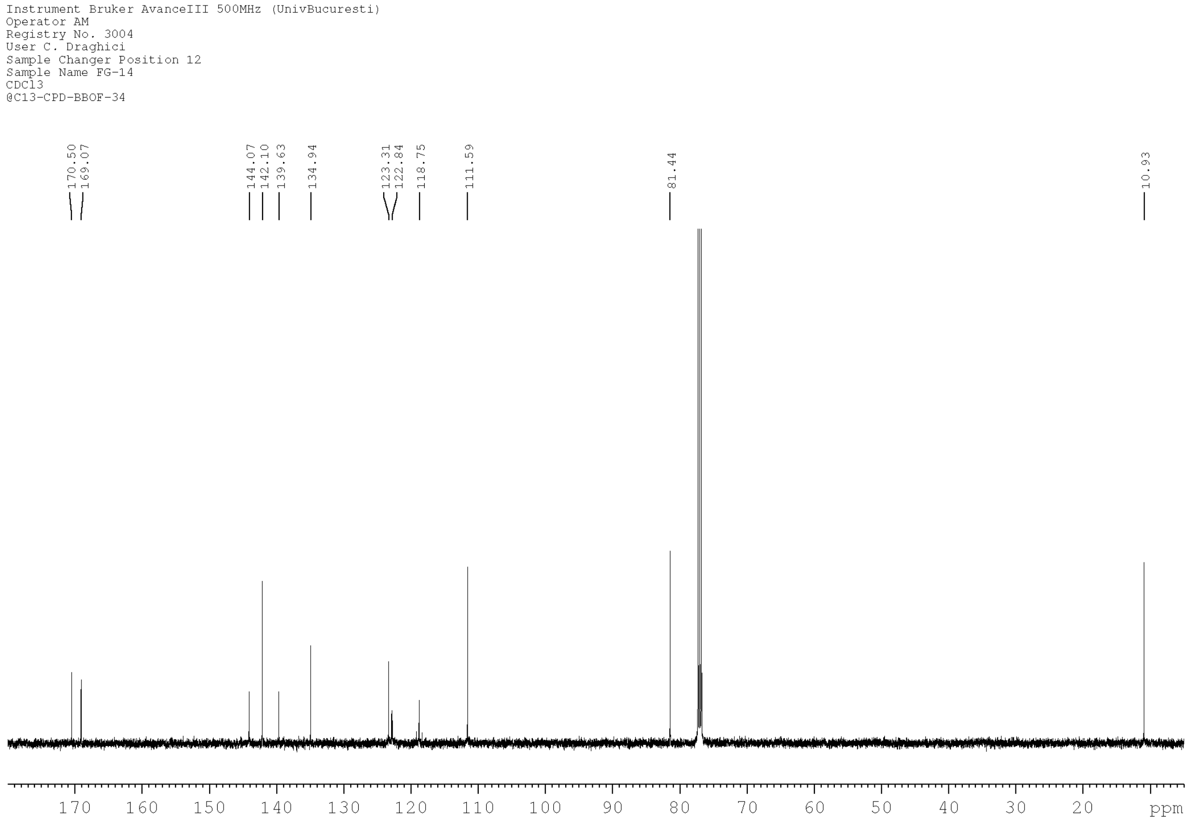

(b)

Figure S7. (a) The ^{1}H NMR spectrum for compound **11** (SL-F6) recorded in $CDCl_3$. (b) The ^{13}C NMR spectrum for compound **11** (SL-F6) recorded in $CDCl_3$.

Crystallographic data

Table S1. Crystal data and details of data collection.

	**7 [SL-FL3]**	**8**
empirical formula	$C_{12}H_9NO_3S$	C_7H_4BrNOS
Fw	247.26	230.08
space group	*I*2/*a*	*C*2/*c*
	28.065(2)	16.8477(14)
b [Å]	4.0890(3)	3.9602(3)
c [Å]	18.9725(13)	23.005(3)
α [°]	90	90
β [°]	94.700(6)	98.437(10)
γ [°]	90	90
V [Å^3]	2169.9(3)	1518.3(3)
Z	8	8
r_{calcd} [g cm^{-3}]	1.514	2.013
Crystal size [mm]	0.35 × 0.05 × 0.05	0.40 × 0.10 × 0.02
T [K]	293	293
μ [mm^{-1}]	0.292	5.620
2Θ range [°]	4.308 to 59.116	3.58 to 57.986
Reflections collected	9289	4178
Independent reflections	2636[R_{int}=0.0413]	1717[R_{int}=0.0368]
Data/restraints/parameters	2636/0/155	1717/0/100
R_1[a]	0.0505	0.0538
wR_2[b]	0.1203	0.1075
GOF[c]	1.029	1.076
Largest diff. peak/hole [e Å^{-3}]	0.24/-0.36	0.34/-0.69
CCDC	2109012	2109011

[a] $R_1 = \Sigma||F_o| - |F_c||/\Sigma|F_o|$. [b] $wR_2 = \{\Sigma[w(F_o^2 - F_c^2)^2]/\Sigma[w(F_o^2)^2]\}^{1/2}$. [c] GOF = $\{\Sigma[w(F_o^2 - F_c^2)^2]/(n - p)\}^{1/2}$, where *n* is the number of reflections and *p* is the total number of parameters refined.

Table S2. Bond distances (Å) and angles (°) of compound **8**

Bond	Distance (Å)	Bond	Distance (Å)	Bonds	Angle (°)	Bonds	Angle (°)
Br1-C3	1.893(5)	C1-C2	1.371(6)	C1-S1-C7	91.4(2)	C3-C4-C5	119.9(4)
S1-C1	1.756(4)	C1-C6	1.391(6)	C7-N1-C6	115.4(4)	C6-C5-C4	118.6(5)
S1-C7	1.778(5)	C2-C3	1.381(6)	C2-C1-S1	128.7(3)	C1-C6-N1	113.3(4)
O1-C7	1.217(6)	C3-C4	1.385(6)	C2-C1-C6	121.1(4)	C5-C6-N1	126.0(4)
N1-C6	1.397(6)	C4-C5	1.388(7)	C6-C1-S1	110.2(4)	C5-C6-C1	120.7(5)
N1-C7	1.358(6)	C5-C6	1.378(6)	C1-C2-C3	118.0(4)	O1-C7-S1	124.4(4)
				C2-C3-Br1	118.6(4)	O1-C7-N1	125.9(5)
				C2-C3-C4	121.7(5)	N1-C7-S1	109.7(4)
				C4-C3-Br1	119.7(4)		

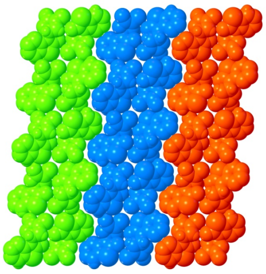

Figure S8. Space filling representation of the crystal structure **7** showing the packing of 2D layers.

Table S3. Bond distances (Å) and angles (°) of compound **7** (SL-F3)

Bond	Distance (Å)	Bonds	Angle (°)	Bonds	Angle (°)
S1-C1	1.746(2)	C1-S1-C7	91.82(11)	O2-C8-C9	104.66(18)
S1-C7	1.764(2)	C11-O2-C8	108.45(16)	N1-C8-C9	117.44(19)
O1-C7	1.212(3)	C6-N1-C8	121.04(18)	C10-C9-C8	109.9(2)
O2-C8	1.437(3)	C7-N1-C6	114.77(19)	C9-C10-C11	107.9(2)
O2-C11	1.374(3)	C7-N1-C8	124.18(19)	C9-C10-C12	131.3(2)
O3-C11	1.196(3)	C2-C1-S1	128.10(19)	C11-C10-C12	120.8(2)
N1-C6	1.409(3)	C2-C1-C6	120.7(2)	O2-C11-C10	108.80(19)
N1-C7	1.383(3)	C6-C1-S1	111.17(17)	O3-C11-O2	120.7(2)
N1-C8	1.443(3)	C3-C2-C1	118.1(2)	O3-C11-C10	130.5(2)
C1-C2	1.379(3)	C4-C3-C2	121.2(2)		
C1-C6	1.393(3)	C3-C4-C5	121.2(2)		
C2-C3	1.379(4)	C6-C5-C4	117.9(2)		
C3-C4	1.375(3)	C1-C6-N1	112.4(2)		
C4-C5	1.383(3)	C5-C6-N1	126.7(2)		
C5-C6	1.379(3)	C5-C6-C1	120.9(2)		
C8-C9	1.491(3)	O1-C7-S1	124.32(19)		
C9-C10	1.320(3)	O1-C7-N1	126.0(2)		
C10-C11	1.469(3)	N1-C7-S1	109.71(16)		
C10-C12	1.492(3)	O2-C8-N1	109.37(16)		
